# Global trends and prospects of ocular biomarkers in Alzheimer’s disease: a bibliometric analysis

**DOI:** 10.3389/fnagi.2025.1528527

**Published:** 2025-03-19

**Authors:** Yufei Shen, Xiaoling Zhang, Congying Xu, Zhuoying Zhu

**Affiliations:** Department of Neurology, The Second Affiliated Hospital of Jiaxing University, Zhejiang, China

**Keywords:** Alzheimer’s disease, CiteSpace, VOSviewer, bibliometric analysis, ocular biomarkers

## Abstract

**Background:**

Alzheimer’s disease (AD) diagnosis necessitates the development of novel biomarkers that ensure high diagnostic accuracy and cost-effectiveness in blood tests. Recent studies have identified a significant association between ocular symptoms and the pathological processes of AD, suggesting the potential for effective ocular biomarkers. This bibliometric analysis aims to explore recent advancements and research trends in ocular biomarkers for the early diagnosis of AD.

**Methods:**

Articles related to AD and ocular biomarkers were retrieved from the Web of Science Core Collection (WoSCC) database. These articles were analyzed using bibliometric tools such as VOSviewer, R-bibliometrix, and CiteSpace.

**Results:**

A total of 623 papers were included in the analysis, revealing a steady increase in publications since 2012. The United States produced the most publications, followed by China and Italy. Notably, authors affiliated with Complutense University of Madrid in Spain and Sapienza University of Rome in Italy made significant contributions, demonstrating robust internal collaborations. The *Journal of Alzheimer’s Disease* published the most articles pertaining to ocular science and neuroscience. Keyword analysis indicates evolving trends in ocular markers for AD from 2005 to 2024, transitioning from diagnostic techniques (e.g., “spectroscopy,” “cerebrospinal fluid”) to pathological mechanisms (e.g., “oxidative stress”) and advanced imaging technologies (e.g., “optical coherence tomography angiography”).

**Conclusion:**

The bibliometric analysis highlights key research hotspots related to ocular markers for AD, documenting the shift from basic diagnostic techniques to advanced imaging methods and the discovery of novel biomarkers. Future research may investigate the potential of Optical Coherence Tomography Angiography, tear component analysis, eye movement assessments, and artificial intelligence to enhance early detection of AD.

## Introduction

Alzheimer’s disease (AD) is a neurodegenerative disorder characterized by a continuous and progressive decline in cognitive function and memory. It has emerged as the leading cause of dementia among individuals aged 65 and older (Klyucherev et al., 2022). The incidence of AD is anticipated to rise progressively with advancing age, presenting significant challenges not only to the health of the elderly population but also to their family members, as well as to the national healthcare system and economy (Yang et al., 2013). The identification of biomarkers that enable the early diagnosis of AD, along with the prompt initiation of interventional therapy, is of paramount importance.

Neurodegeneration and brain atrophy in AD are correlated with the presence of neuritic plaques and fibrillary tangles, which are characterized by the deposition of amyloid-β (Aβ) and the accumulation of hyperphosphorylated tau protein (Ballard et al., 2011). Positron emission tomography (PET) is highly regarded for its sensitivity and low variability, making it effective for the early detection of AD in pre-symptomatic patients or high-risk populations, although it incurs a significant financial cost (Ossenkoppele and Hansson, 2021). Cerebrospinal fluid (CSF) biomarkers demonstrate high diagnostic accuracy and can identify abnormalities before PET imaging, thus enabling earlier diagnosis of AD; however, these methods necessitate invasive sampling and can be time-consuming (Palmqvist et al., 2017). In comparison, measuring tau levels in plasma is less invasive, more accessible, and feasible for broad implementation in primary healthcare settings, making it a promising preliminary approach for screening AD in high-risk populations (Klyucherev et al., 2022). However, differentiating AD from other neurodegenerative disorders using plasma tests at the preclinical stage remains a substantial challenge. The retina, as the only extra-cranial extension of the central nervous system, presents a potential biomarker for pathological changes in the brain associated with neurodegenerative diseases (Koronyo-Hamaoui et al., 2011). Research has established increased Aβ deposition in the retinas of patients with AD compared to cognitively normal individuals, along with a close association between Aβ deposition and heightened retinal microglial proliferation. Furthermore, there is a relationship between microglial proliferation and tissue atrophy in retinopathy, influenced by the severity of neuropathological changes (Koronyo et al., 2023). Recent studies have suggested that retinopathy may manifest during the early asymptomatic phase of AD, indicating that ocular markers could be valuable new indicators for addressing previously recognized deficiencies (den Haan et al., 2017). Optical coherence tomography (OCT) is an essential diagnostic modality in the field of ophthalmology, providing high-resolution imaging of the retina as well as precise measurements of retinal nerve fiber layer (RNFL) thickness. Evidence indicates that individuals diagnosed with AD often exhibit notable thinning of the RNFL, particularly in specific retinal quadrants(Kesler et al., 2011; van de Kreeke et al., 2019). Additionally, numerous investigations have documented other ocular degenerations in AD patients, underscoring the connection between ocular conditions and disease progression (Frost et al., 2013; Chang et al., 2014; La Morgia et al., 2016; Uchida et al., 2018). Given that AD is known to be associated with vascular changes (Jellinger, 2002), optical coherence tomography angiography (OCTA) — an advanced form of OCT that generates detailed images of the retinal vascular network without the use of contrast agents — could serve as a valuable early biomarker. Specific protein enrichment in tear fluid (Kenny et al., 2019), melanopsin retinal ganglion cell degeneration (La Morgia et al., 2016), and saccadic eye movement parameters (Opwonya et al., 2022) also represent valuable diagnostic tools for AD, providing accessible, non-invasive, and sensitive biomarkers.

The recent increase in literature concerning ocular biomarkers associated with AD has engendered confusion regarding the predominant research areas and emerging trends in this field. To elucidate these complexities, we conducted a comprehensive bibliometric analysis of ocular biomarkers utilized in the detection of AD. This analysis assessed both the quantity and quality of publications, along with contributions from various countries, institutions, journals, authors, and keywords. By revealing the distribution, linkages, and clustering of ocular markers used in the detection of AD, we aim to fill gaps in the existing literature by synthesizing findings and trends, providing a detailed overview of the current understanding of ocular markers for early AD detection, and suggesting potential directions for future research. The recent increase in literature on ocular biomarkers related to AD has engendered some confusion about the key research areas and emerging trends in this field. To clarify this complexity, we conducted a comprehensive bibliometric analysis of ocular biomarkers used in AD detection. This analysis assessed both the quantity and quality of publications, as well as the contributions from various countries, institutions, journals, authors, and keywords(Donthu et al., 2021). By mapping the distribution, connections, and clustering of ocular markers for AD detection, we aim to fill gaps in the existing literature. Our synthesis of findings and trends will provide a detailed overview of the current understanding of ocular markers for early AD detection and suggest potential directions for future research.

## Materials and methods

### Search strategies and data collection

We conducted a literature search using the Web of Science Core Collection (WoSCC), known for its high-quality data and robust citation analysis tools. The search formula was: (TS = (((Ocular OR eye OR optic* OR “conjunctival” OR “conjunctivitis” OR “conjunctiv*” OR “ophthalm*” OR tear) AND biomarker*) OR “Ocular biomarker”)) AND TS = (Alzheimer* OR “Alzheimer disease” OR “Alzheimer’s disease” OR “Senile Dementia”; Costanzo et al., 2023; Kim et al., 2023). We restricted our search to English-language articles only. To minimize variations owing to database updates, we performed the literature retrieval on August 7, 2024. All relevant information, including publications and citation counts, titles, author details, institutions, countries/regions, keywords, and journals, was collected for further bibliometric analysis.

### Statistical analysis

Microsoft Excel (version 2016), R-bibliometric (version 4.3.3), VOSviewer (version 1.6.20), and CiteSpace (version 6.3.R1) were used for data analysis and visualization. Excel was used for data collection, table generation, calculation of key indicators, and analysis of annual publication trends. R-bibliometric enabled the visualization of published article volumes and performance analysis(Donthu et al., 2021). The H-index, a crucial metric for evaluating academic contributions, was obtained from WoSCC to quantify the academic impact of countries/regions, institutions, and journals (Tahamtan et al., 2016). VOSviewer was employed to visualize collaborative networks among countries, institutions, and authors, along with the co-occurrence and coupling networks of journals and keyword co-occurrence networks. In these visualizations, node sizes represented the number of publications, while links indicated co-authorships, with their thickness reflecting the strength of collaboration. Different colors represented research clusters, and total link strength in collaboration networks measured the frequency of co-authorship, highlighting the extent of collaborative research (Liu et al., 2024). CiteSpace was utilized for keyword burst analyses, with time slicing configured to one-year intervals. The threshold for the top N keywords per segment was established at 5, and both the Pathfinder method and pruning of the merged network were employed (Sabe et al., 2022). This methodology produced a keyword timeline centered on ocular biomarkers and AD, accurately reflecting the specific parameter settings for each node.

## Results

### Overview of publications

**Figure 1:**
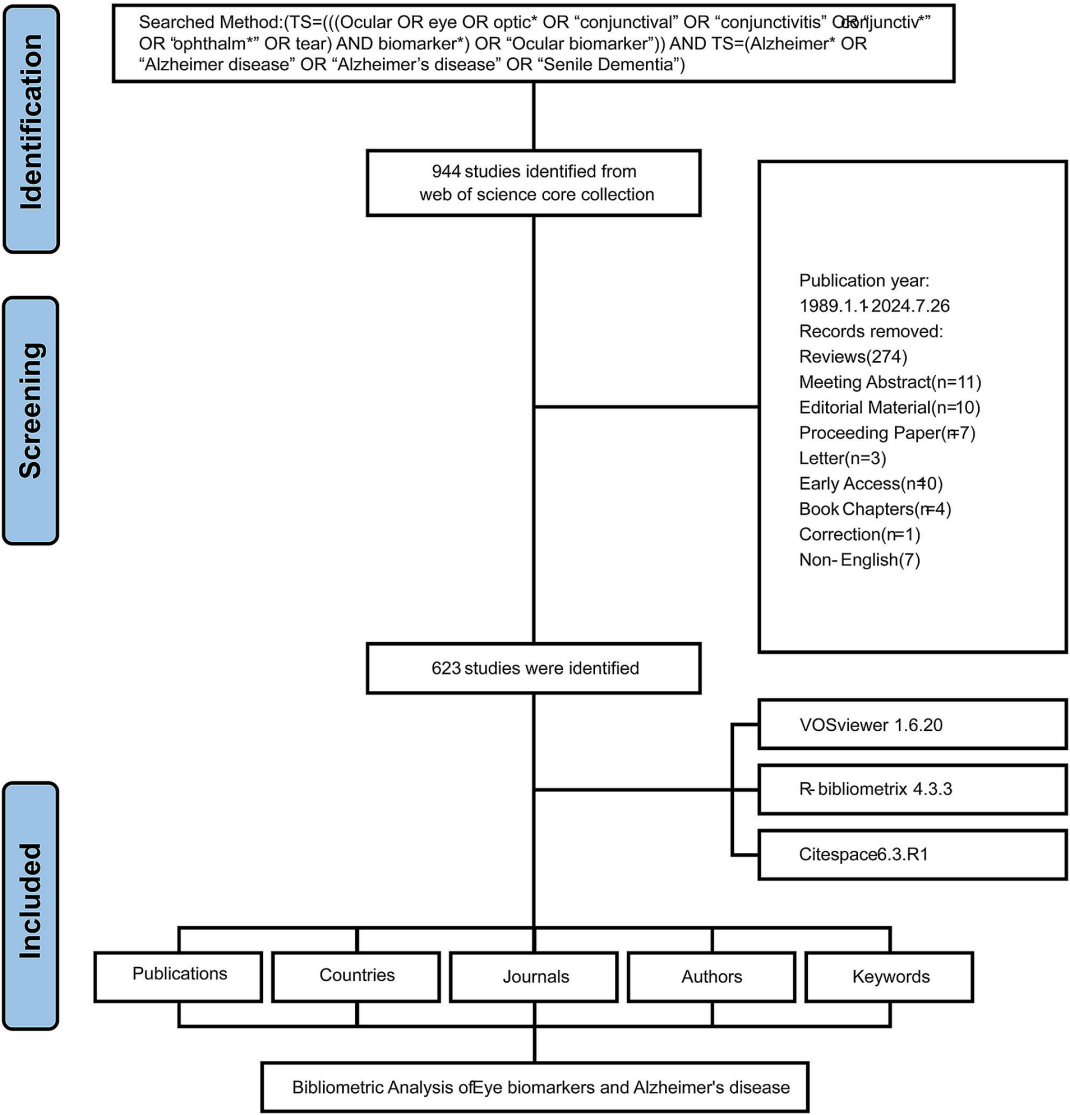
Flowchart of the literature screening process.

**Figure 2:**
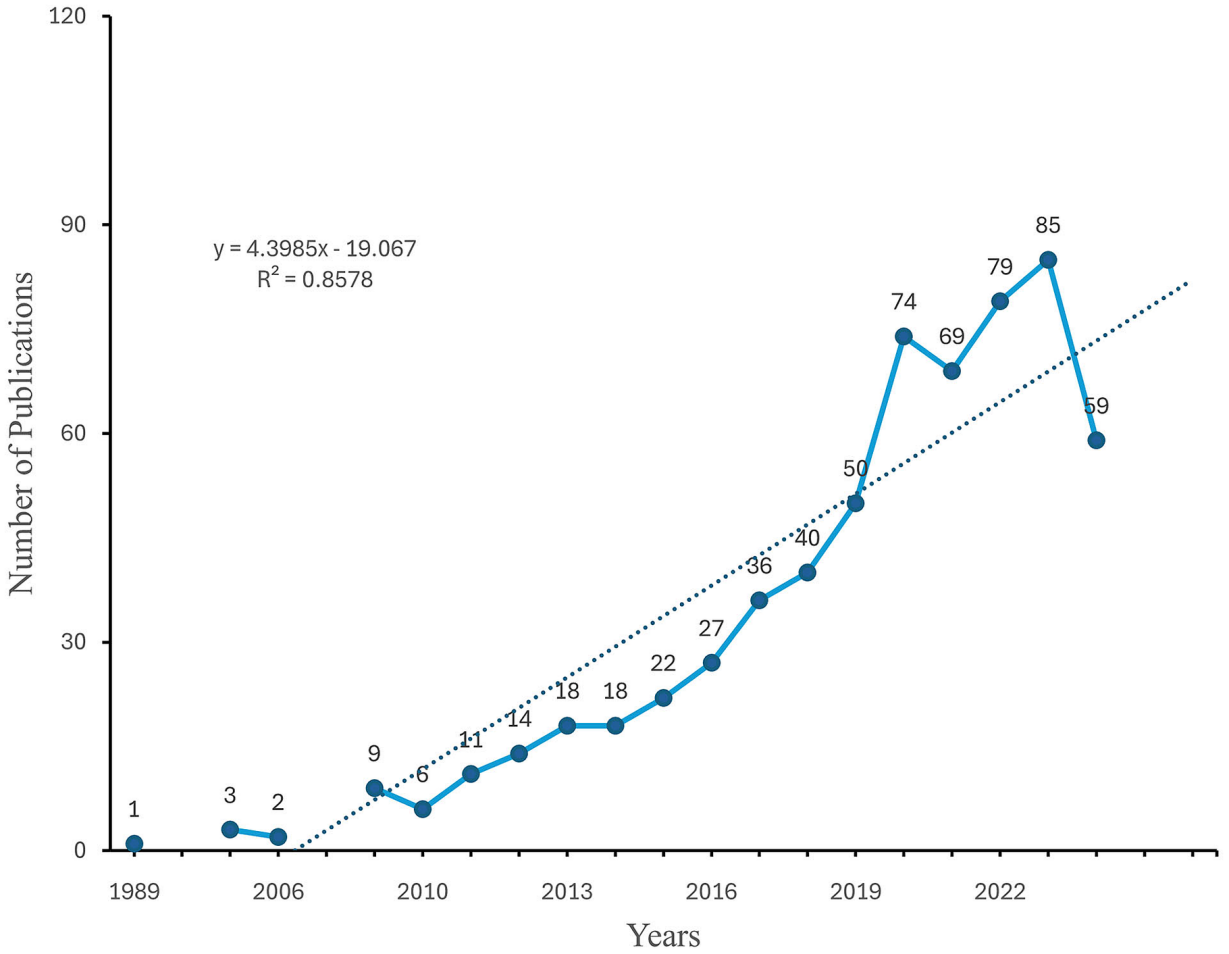
Annual number of publications.

A total of 623 eligible publications were analyzed, as illustrated in the data screening flowchart in Figure 1. These publications, sourced from 267 journals, span the years 1989 to 2024 and include contributions from 4,564 authors, five of whom are independent authors. On average, each publication featured 8.84 co-authors, with international collaborations accounting for 32.42% of the total authorship. The average age of the publications was 4.72 years, and they received an average of 28.05 citations. Our analysis identified 1,543 unique keywords. In 1989, the inaugural year of the publication period, only one article was published. Following a 15-year hiatus, the number of published articles began to increase steadily in 2005. Figure 2 illustrated a significant rise in publications, with the total increasing from 50 in 2019 to 74 in 2020, and peaking at 85 publications in 2023. A simple linear regression analysis produced the equation: y = 4.3985x − 19.067, with a coefficient of determination of 0.8526. This finding indicated promising growth in this research area.

### Analysis of countries

**Figure 3:**
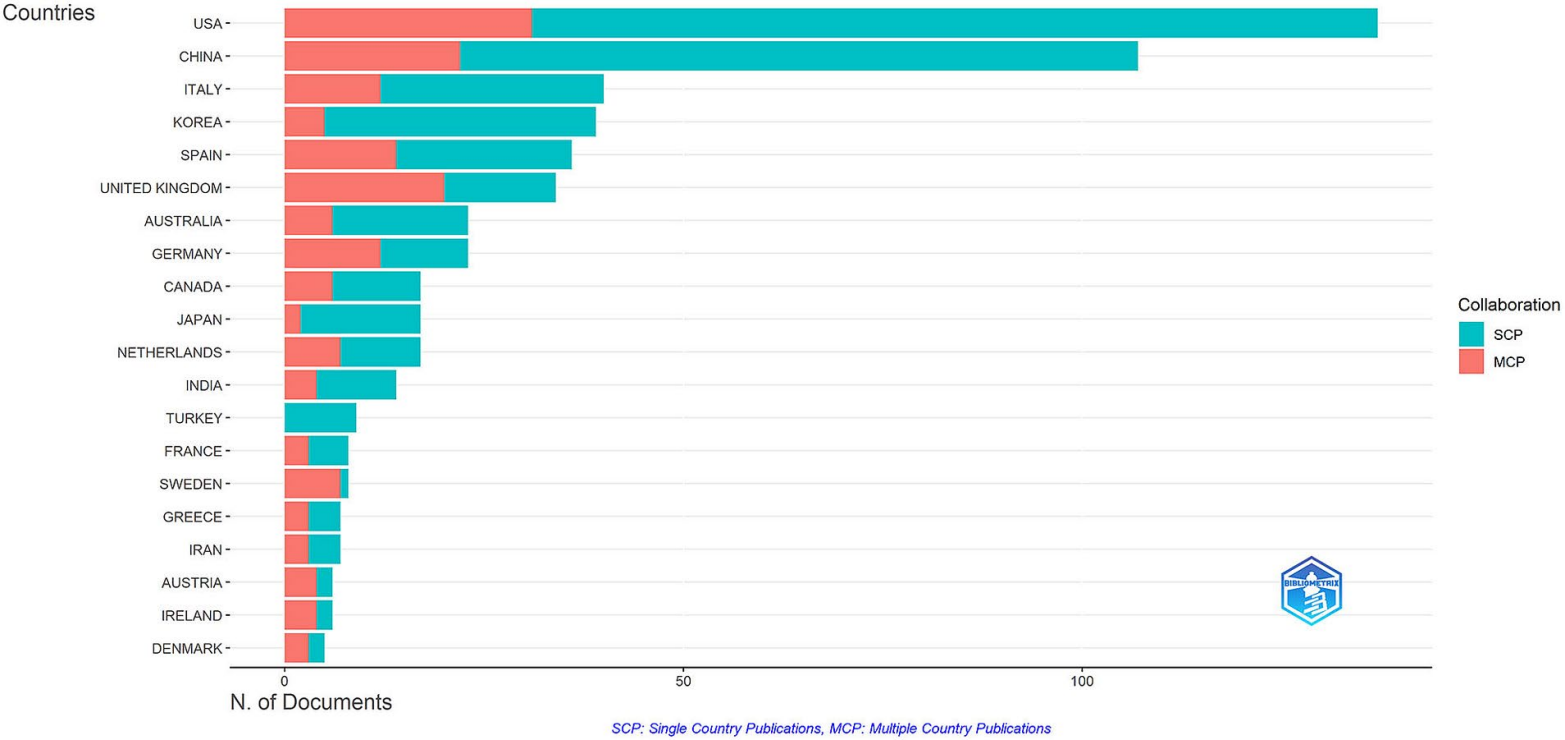
Distribution of corresponding author’s publications by country (R bibliometrix).

**Figure 4:**
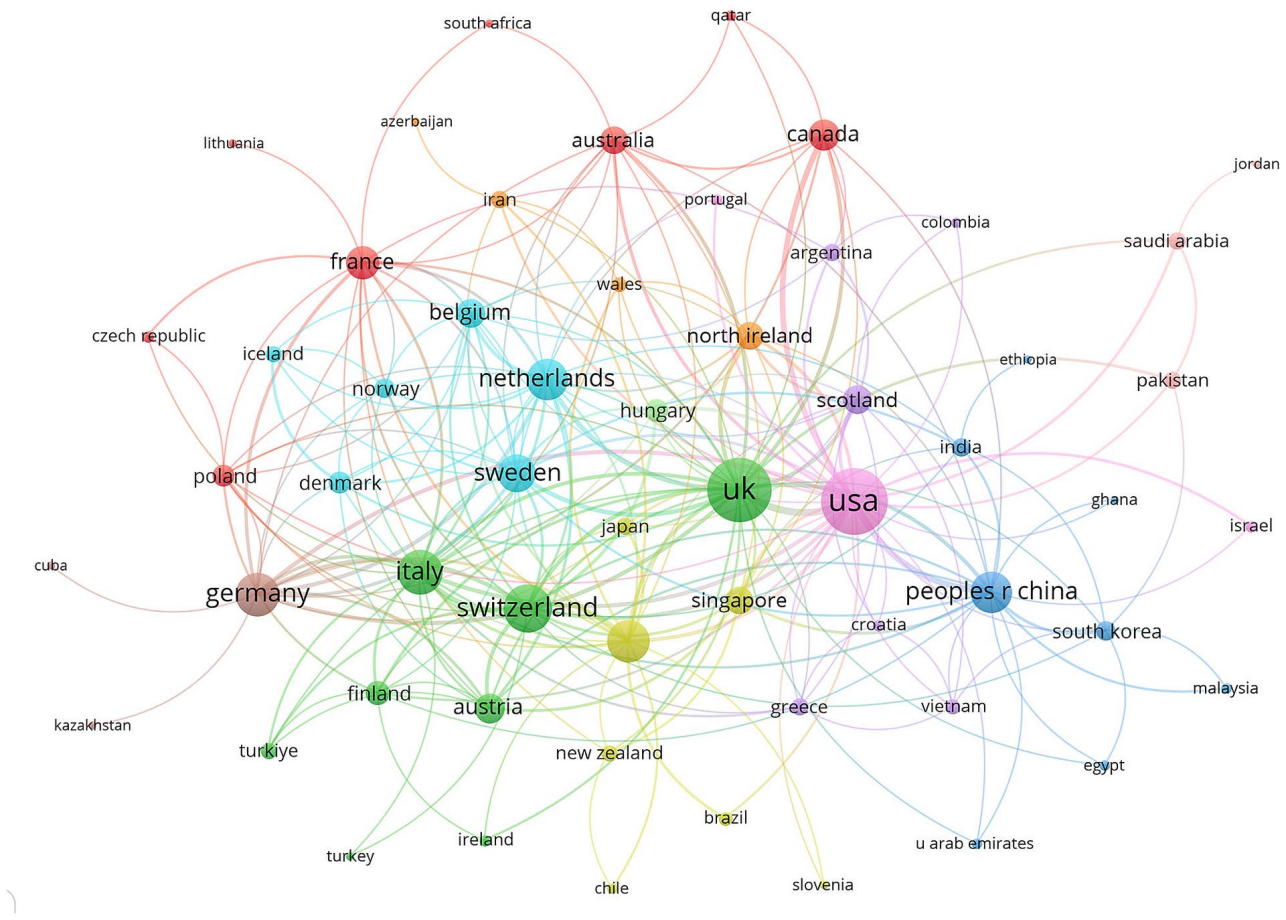
Visualization map depicting the collaboration among different countries.

As shown in Table 1, most of the published literature on ocular biomarkers and AD originated from the United States (137 publications), China (107 publications), and Italy (40 publications), collectively accounting for 45.59% of all studies. The United States led with a total of 716 publications and 3,990 citations, followed by China with 427 publications and 1,980 citations, underscoring their substantial influence in this research area (see Table 1 and Figure 3). Additionally, Sweden boasted the highest Proportion of Multiple Country Publications (MCP_Ratio) at 87.50, indicating strong collaborative efforts. Among the top 20 countries, Germany had the highest average citations at 62.0, closely followed by Turkey at 60.3, suggesting that Turkey produces high-quality research despite its limited international collaboration. Of the 61 countries and regions involved in international collaborations with at least one published article, the United States demonstrated the strongest total link strength (119), followed by the United Kingdom (112) and Switzerland (63; see Figure 4). The interconnected clusters illustrated in Figure 4 reflect a significant level of global collaboration within this field.

**Table 1:** Publication and citation profiles of leading countries.

Country	Articles	Freq	MCP_Ratio	TP	TP_rank	TC	TC_rank	Average Citations
USA	137	21.99	22.63	716	1	3,990	1	29.1
China	107	17.17	20.56	427	2	1,980	2	18.5
Italy	40	6.42	30.00	204	4	991	5	24.8
Korea	39	6.26	12.82	170	6	528	11	13.5
Spain	36	5.78	38.89	224	3	980	6	27.2
United kingdom	34	5.46	58.82	204	5	1,463	3	43
Australia	23	3.69	26.09	159	7	889	7	38.7
Germany	23	3.69	52.17	102	10	1,427	4	62
Canada	17	2.73	35.29	104	9	886	8	52.1
Japan	17	2.73	11.76	84	11	291	14	17.1
Netherlands	17	2.73	41.18	111	8	831	9	48.9
India	14	2.25	28.57	46	15	222	15	15.9
Turkey	9	1.44	0.00	32	20	543	10	60.3
France	8	1.28	37.50	57	13	293	13	36.6
Sweden	8	1.28	87.50	43	16	210	16	26.2
Greece	7	1.12	42.86	24	24	82	26	11.7
Iran	7	1.12	42.86	31	21	155	18	22.1
Austria	6	0.96	66.67	21	25	176	17	29.3
Ireland	6	0.96	66.67	26	22	305	12	50.8
Denmark	5	0.80	60.00	25	23	115	22	23

Articles: Publications of Corresponding Authors only. Freq: Frequence of Total Publications. MCP_Ratio: Proportion of Multiple Country Publications. TP: Total Publications. TP_rank: Rank of Total Publications. TC: Total Citations. TC_rank: Rank of Total Citations. Average Citations: The average number of citations.

### Analysis of institutions

**Figure 5:**
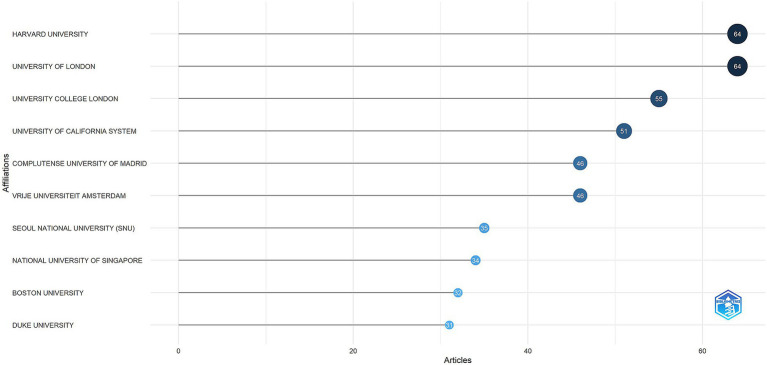
Top 10 institutions by article count and rank (R bibliometrix).

**Figure 6:**
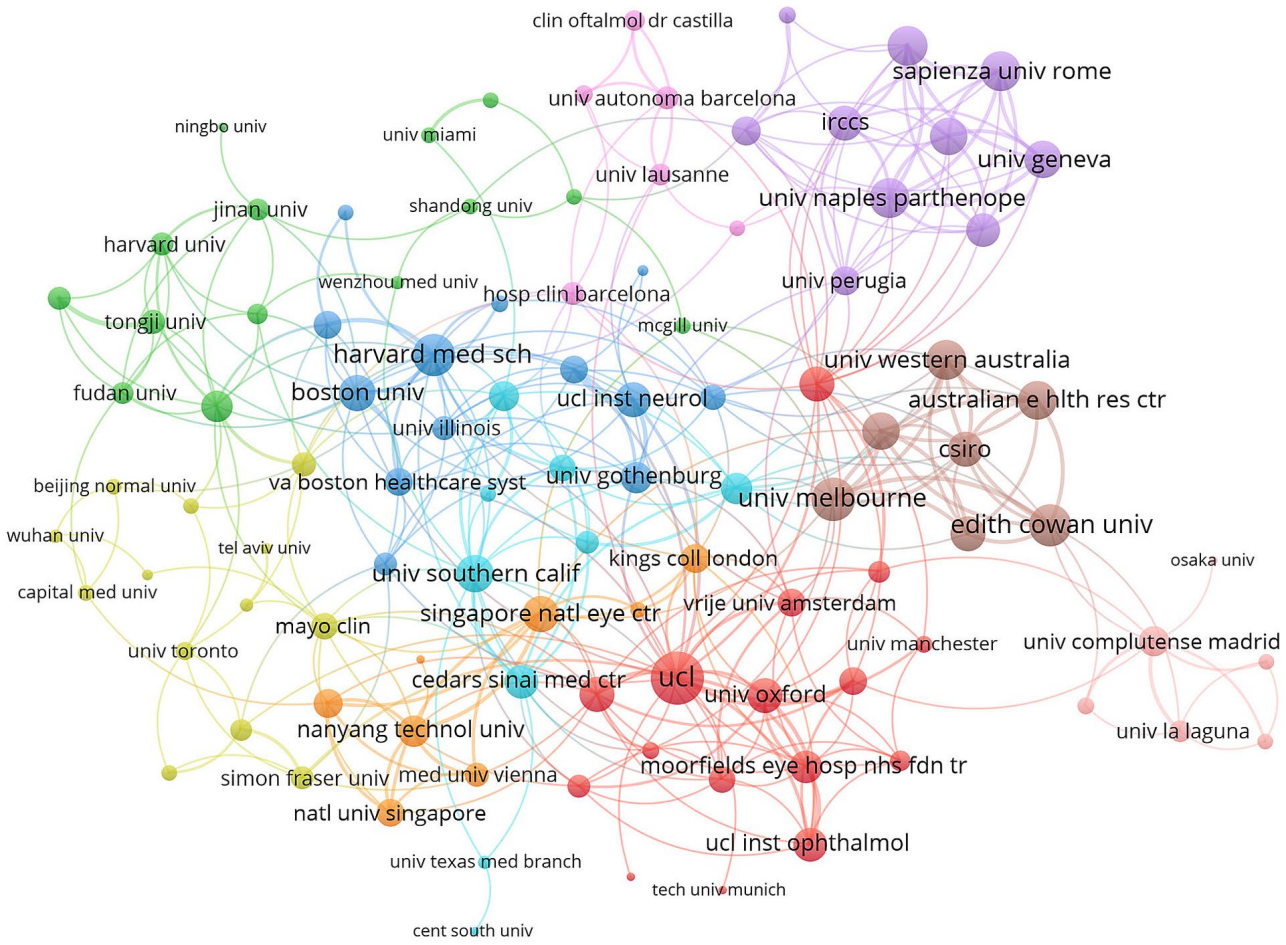
Visualization map depicting the collaboration among different institutions.

The top 10 institutions with the highest number of publications were illustrated in Figure 5. Harvard University and the University of London were tied for first place, each contributing 64 publications, followed by University College London with 55. The international collaboration network among 113 institutions that produced a minimum of four articles was depicted in Figure 6. Ten clusters were identified, indicating substantial connections among the institutions. The red cluster, representing institutions from the United Kingdom, was centered around University College London and the University of Oxford, with University College London exhibiting the highest number of collaborations (46). The institutions within the purple cluster, which included Sapienza University of Rome and the University of Perugia, are predominantly from Italy. The brown cluster, featuring the University of Western Australia and the University of Melbourne, was primarily composed of Australian institutions. The blue cluster, which mainly consisted of institutions from the United States, included Harvard Medical School and Boston University. The green cluster was largely comprised of Chinese institutions, such as Tongji University and Fudan University. This visualization suggested that institutions from the same country or region tend to aggregate within the same cluster.

### Analysis of authors

**Figure 7:**
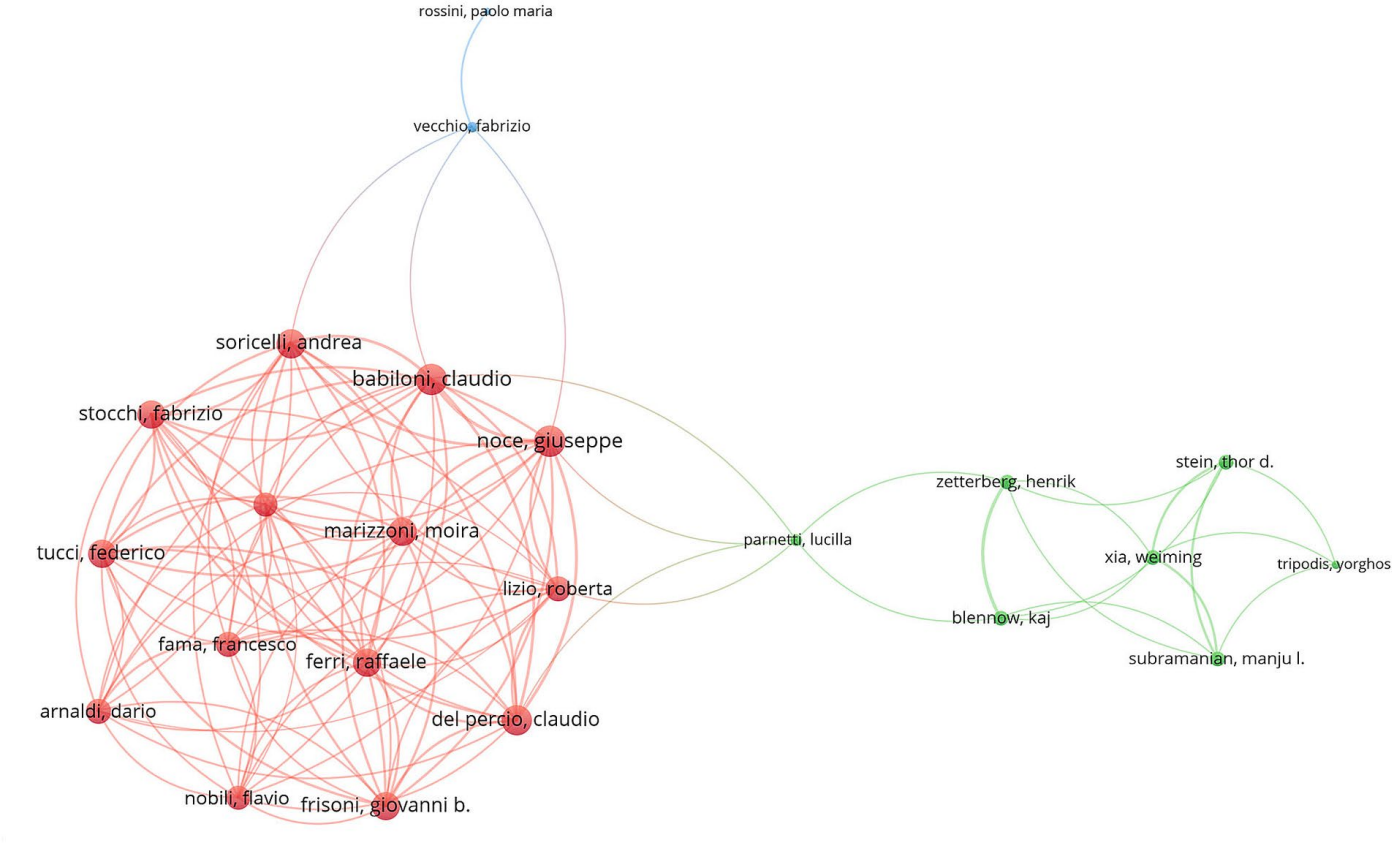
Visualization map depicting the collaboration among different authors.

A total of 4,564 authors contributed to publications in this field, with the top 20 most cited authors listed in Table 2. Verbraak Frank D. ranked first in both TP (9) and TC (53), underscoring his significant authority in this domain. Despite Ramirez Jose M. ranked second in TP (8), he was positioned 34^th^ in TC (30). Additionally, Figure 7 visualizes the co-authorship network of 150 authors with at least three articles. Ramirez Jose M. (50) and Salobrar-Garcia Elena (50) had the highest number of international collaborations, followed by De Hoz Rosa (43), Salazar Juan J. (43), and Verbraak Frank J (43). Three clusters were depicted, with the red cluster (containing Babiloni Claudio and Noce Giuseppe) being the largest. The green cluster was relatively small, containing Zetterberg Henrik and Xia Weiming, while the blue cluster was the smallest, with only two authors.

**Table 2:** Publication and citation profiles of high-impact authors.

Authors	H_index	g-index	m-index	PY_start	TP	TP_Frac	TP_rank	TC	TC_rank
Masters Colin L.	7	7	0.500	2011	7	0.649	6	35	25
Verbraak Frank D.	7	9	1.000	2018	9	0.716	1	53	1
Kanagasingam Yogesan	6	6	0.500	2013	6	0.555	9	17	217
Ramirez Jose M.	6	8	0.600	2015	8	0.744	2	30	34
Salobrar-Garcia Elena	6	8	0.600	2015	8	0.744	3	30	35
Visser Pieter Jelle	6	7	0.857	2018	7	0.572	7	29	52
Blennow Kaj	5	5	0.556	2016	5	0.321	16	6	507
De Hoz Rosa	5	7	0.500	2015	7	0.661	4	24	84
Den Braber Anouk	5	5	0.714	2018	5	0.347	22	24	85
Frost Shaun	5	5	0.417	2013	5	0.455	25	11	308
Konijnenberg Elles	5	5	0.714	2018	5	0.347	27	24	87
Lengyel Imre	5	7	0.714	2018	7	0.636	5	21	112
Li Chunbo	5	5	0.417	2013	5	0.581	29	41	11
Lopez-Cuenca Ines	5	5	1.000	2020	5	0.403	30	11	313
Maestu Fernando	5	6	0.714	2018	6	0.762	10	7	469
Martins Ralph N.	5	5	0.417	2013	5	0.455	32	37	24
Salazar Juan J.	5	6	0.500	2015	6	0.494	12	23	96
Scheltens Philip	5	6	0.313	2009	6	0.563	13	39	16
Shen Yuan	5	5	0.417	2013	5	0.581	35	41	13
Shi Zhongyong	5	5	0.417	2013	5	0.581	36	41	14

H_index: The h-index of the journal, which measures both the productivity and citation impact of the publications. g_index: The g-index of the journal, which gives more weight to highly-cited articles. m_index: The m-index of the journal, which is the h-index divided by the number of years since the first published paper. TP: Total Publications. TP_rank: Rank of Total Publications. TC: Total Citations. TC_rank: Rank of Total Citations. Average Citations: The average number of citations per publication. PY_start: Publication Year Start, indicating the year the journal started publication.

### Analysis of journals

**Figure 8:**
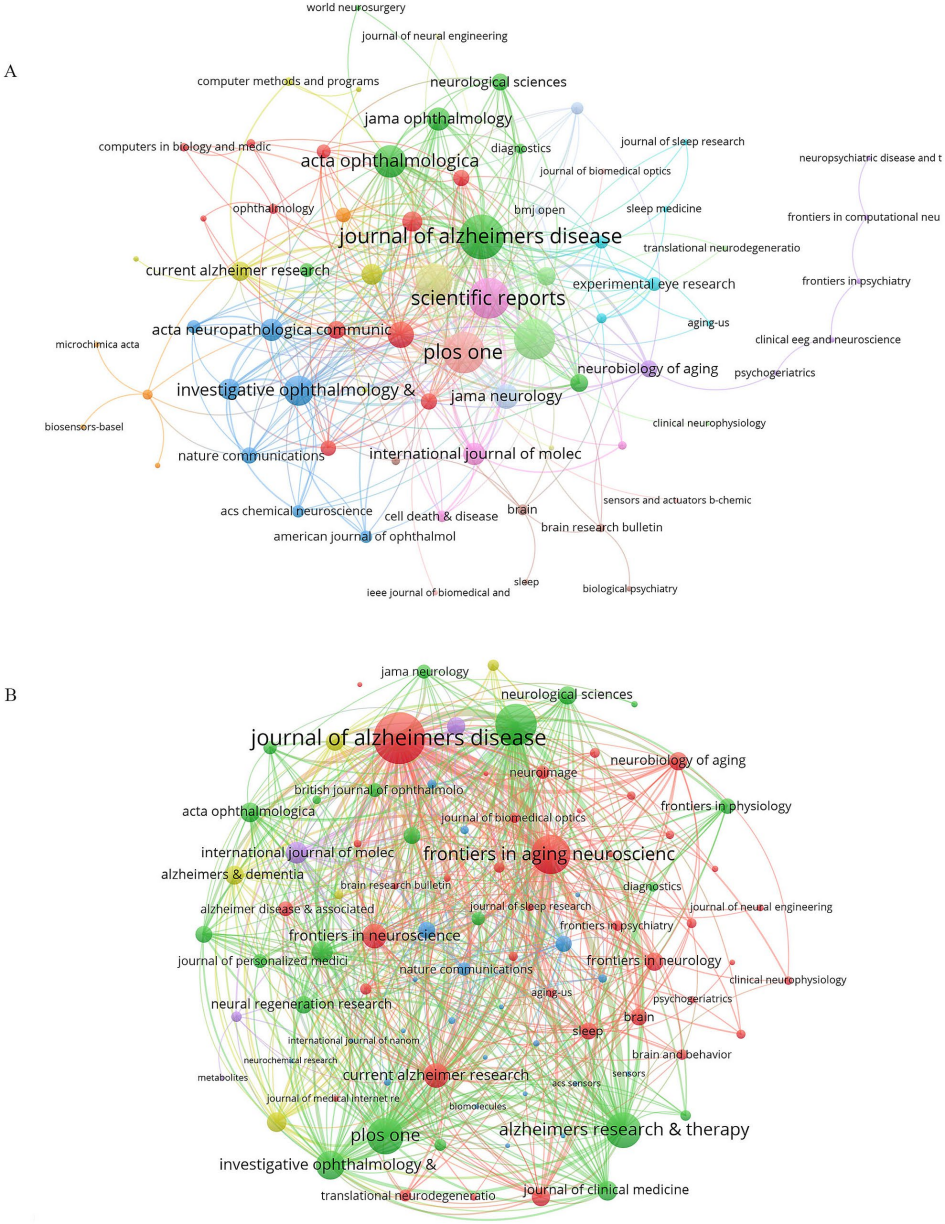
**(A)** Co-occurrence Network of Journals, **(B)** Coupling Network of Journals.

Details on the 20 most prolific journals, ranked by H-index, with the *Journal of Alzheimer’s Disease* taking the top spot, were provided in Table 3. These journals produced 248 publications, representing 39.81% of all retrieved studies. *Biosensors and Bioelectronics* had the highest Impact Factor (IF = 10.7), with *Brain* closely followed (IF = 35.5), highlighting their significant impact. Notably, most of these journals belonged to the Q1 and Q2 quartiles. Moreover, the *Journal of Alzheimer’s Disease* ranked first in TP (42) and third in TC (827), underscoring its influence in this field. Despite fewer TP (18), *Investigative Ophthalmology and Visual Science* (914), signifying the high quality of its publications. Figure 8a depicts the co-occurrence network of 92 journals with at least 2 occurrences, with the *Journal of Alzheimers Disease* (166) having the highest total link strength, followed by *Plos One* (115) and *Alzheimer’s Research and Therapy* (113), reflecting their central role in connecting with other publications. As for the coupling network analysis (Figure 8b), 92 journals were included with at least 2 couples. *The Journal of Alzheimers Disease* (10,956) ranked first, followed by *Scientific Reports* (6,951) and *Frontiers in Aging Neuroscience* (6,326), emphasizing their central role in addressing common research topics.

**Table 3:** Bibliometric indicators of high-impact journals.

Journal	H_index	IF	JCR_Quartile	PY_start	TP	TP_rank	TC	TC_rank
Journal of Alzheimers Disease	17	3.4	Q2	2010	42	1	827	3
Investigative Ophthalmology and Visual Science	14	5	Q1	2006	18	5	914	1
PLoS One	14	2.9	Q1	2012	20	4	650	5
Scientific Reports	14	3.8	Q1	2016	28	2	377	11
Alzheimer’s Research and Therapy	9	7.9	Q1	2017	14	6	193	30
Frontiers in Aging Neuroscience	9	4.1	Q2	2013	22	3	217	22
Brain	8	10.6	Q1	2010	10	7	509	8
Biosensors and Bioelectronics	7	10.7	Q1	2010	7	16	195	28
Acta Ophthalmologica	6	3	Q1	2016	7	15	168	36
Current Alzheimer Research	6	1.8	Q4	2013	8	11	130	46
Frontiers in Neuroscience	6	3.2	Q2	2018	10	9	178	34
Sleep	6	5.3	Q1	2016	8	14	195	29
ACS Chemical Neuroscience	5	4.1	Q2	2015	6	17	68	88
Analytical Chemistry	5	6.7	Q1	2015	5	22	159	38
Experimental Eye Research	5	3	Q1	2015	8	12	158	40
Frontiers in Neurology	5	2.7	Q3	2013	10	8	170	35
JAMA Ophthalmology	5	7.8	Q1	2021	5	23	97	63
Neurobiology of Aging	5	3.7	Q2	2006	8	13	615	6
Alzheimer’s and Dementia	4	13	Q1	2021	8	10	799	4
British Journal of Ophthalmology	4	3.7	Q1	2009	4	29	200	27

H_index: The h-index of the journal, which measures both the productivity and citation impact of the publications. IF: Impact Factor, indicating the average number of citations to recent articles published in the journal. JCR_Quartile: The quartile ranking of the journal in the Journal Citation Reports, indicating the journal’s ranking relative to others in the same field (Q1: top 25%, Q2: 25–50%, Q3: 50–75%, Q4: bottom 25%). TP: Total Publications. TP_rank: Rank of Total Publications. TC: Total Citations. TC_rank: Rank of Total Citations. Average Citations: The average number of citations per publication. PY_start: Publication Year Start, indicating the year the journal started publication.

### Analysis of keywords

**Figure 9:**
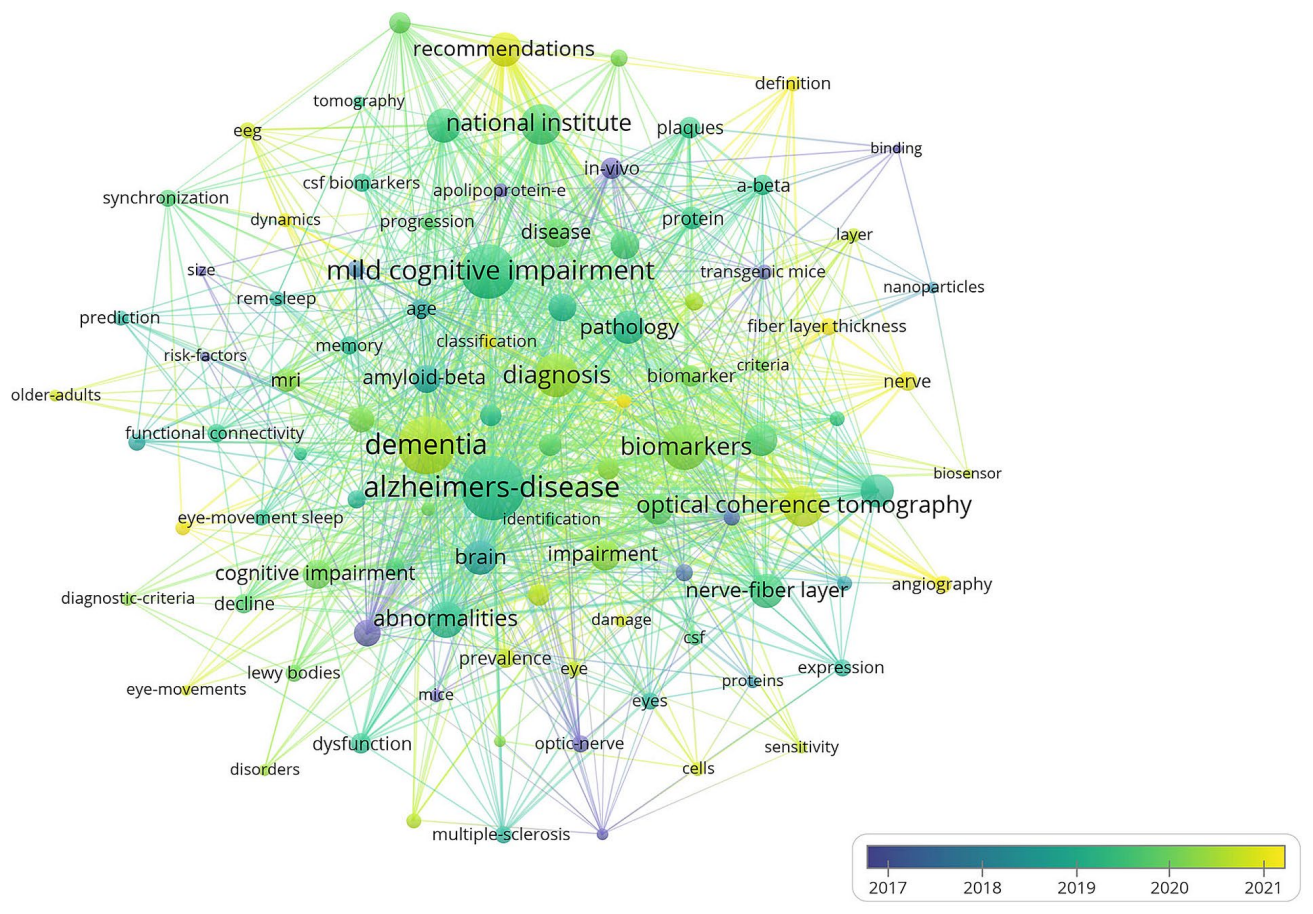
Visual analysis of keyword co-occurrence network analysis.

Keyword co-occurrence analysis explores the relationships between frequently paired keywords revealing connections across various topics and uncovering the underlying structure of knowledge within a field. From 623 articles 98 keywords with at least 8 occurrences were obtained. “Alzheimers-disease” had the highest total link strength (727) with 244 occurrences followed by “dementia” (578) and “mild cognitive impairment” (514) indicating their strong associations with other terms (Figure 9). More recent keywords such as “recommendations” and “definition” (highlighted in yellow) have begun replacing older terms like “national institute” and “mild cognitive impairment” (in green) and earlier terms like “in-vivo” and “binding” (in purple). This shift demonstrates the evolving research focus over the years. Six clusters were detected with cluster 1 (including 32 keywords) the biggest. Cluster 1 centered around “biomarkers” “biomarker” and “nanoparticles.” While cluster 2 (20) contained “mild cognitive impairment” and “national institute” with cluster 3 (17) followed closely including “Alzheimer’s-disease” and “dementia.” Moreover cluster 4 (11) cluster 5 (9) and cluster 6 (9) were relatively small comprised of “nerve” “definition” and “atrophy” separately.

**Figure 10:**
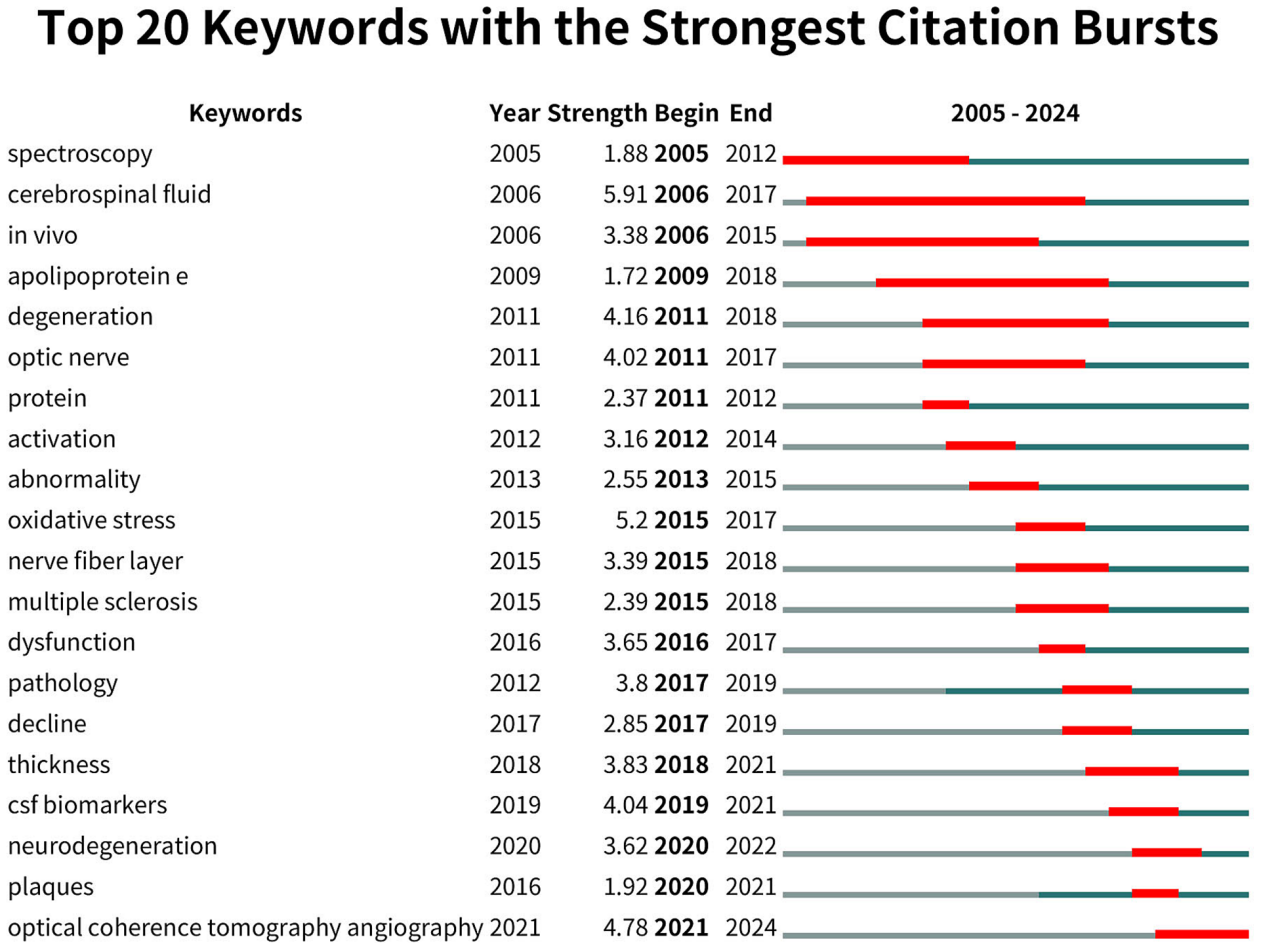
Top 20 keywords with the strongest citation bursts (CiteSpace).

The top 20 terms with the most significant citation bursts related to ocular biomarkers and AD from 2005 to 2024 were illustrated in Figure 10. The grey lines represented the overall timespan, while the red lines indicated the periods of citation bursts. The term “cerebrospinal fluid” (5.91) shows the largest citation burst, followed closely by “optical coherence tomography angiography” (4.78). In 2011, the terms “optic nerve” and “protein” marked the beginning of a new research trend, which has evolved into various topics in the years since. Since 2018, terms such as “thickness,” “csf biomarkers,” “neurodegeneration,” and “plaques,” and “optical coherence tomography angiography” have gained increasing attention and continue to be central to ongoing research, signaling promising advancements in the field (Table 4).

**Table 4:** Summary of specific ocular biomarkers and their corresponding tissues.

Corresponding tissues	Specific biomarkers	Reference
Cornea	Branch density	Romaus-Sanjurjo et al. (2022); Hussain et al. (2023)
Cornea	Fiber length	Romaus-Sanjurjo et al. (2022); Hussain et al. (2023)
Cornea	Corneal nerve fiber density	Romaus-Sanjurjo et al. (2022); Hussain et al. (2023)
Lens	Amyloid-β peptide	Romaus-Sanjurjo et al. (2022)
Retina	Ganglion cells and inner plexiform Layer	Jin et al. (2025)
Retina	Inner nuclear layer	Jin et al. (2025)
Retina	Outer nuclear layer	Jin et al. (2025)
Retina	Outer plexiform layer	Jin et al. (2025)
Retina	Retinal pigment epithelium	Bai et al. (2022); Jin et al. (2025)
Retina	Drusus deposits	Klyucherev et al. (2022)
Retina	Glial fibrillary acidic protein	Bai et al. (2022); Costanzo et al. (2023)
Retina	Macular	Hussain et al. (2023)
Retina	Müller and microglial cells	Hussain et al. (2023)
Retina	Myoid and ellipsoid zone	Jin et al. (2025)
Retina	Outer segment of photoreceptor	Jin et al. (2025)
Retina	Parietal cortex	Klyucherev et al. (2022)
Retina	Peripheral retinal ganglion cell layers	Klyucherev et al. (2022)
Retina	Retinal ganglion cell layer	(Costanzo et al., 2023; Hussain et al., 2023)
Retina	Retinal thickness	Klyucherev et al. (2022)
Retina	The retinal nerve fiber layer	Klyucherev et al. (2022)
Retina	Retinal deposits of Aβ and tau	Bai et al. (2022); Klyucherev et al. (2022); Romaus-Sanjurjo et al. (2022); Costanzo et al. (2023); Hussain et al. (2023); Jin et al. (2025)
Retina	Retinal vascular	Romaus-Sanjurjo et al. (2022); Costanzo et al. (2023)
Tear Fluid	Lysozyme, lipocalin-1, lacritin and dermcidin	Romaus-Sanjurjo et al. (2022); Hussain et al. (2023)
Tear Fluid	MicroRNA-200b-5p and elf4E as biomarkers	Romaus-Sanjurjo et al. (2022); Hussain et al. (2023)
Tear Fluid	Tau protein and amyloid-β peptide	Romaus-Sanjurjo et al. (2022); Hussain et al. (2023)
Vitreous Humor	Neurofilament light chain	Romaus-Sanjurjo et al. (2022)
Vitreous Humor	Soluble amyloid precursor protein	Romaus-Sanjurjo et al. (2022)

## Discussion

### General information

The investigation into ocular markers for the early asymptomatic detection of AD commenced in 1989. However, a notable increase in the published literature was not evident until 2012, with a more pronounced surge occurring in 2019. An analysis of the geographical distribution of this research indicates that the United States has emerged as the leading nation in the study of ocular markers and AD diagnostics, producing the highest volume of published studies and maintaining significant collaborative ties with other countries. Notably, the rate of international collaboration within the United States remains relatively low, suggesting a preference for domestic partnerships. Furthermore, a review of pertinent journals focused on ocular markers and AD reveals that researchers are increasingly specializing in fields such as neuroscience, ophthalmology, and gerontology, particularly in relation to AD. Prominent journals in this domain, including the *Journal of Alzheimer’s Disease*, *Plos One*, and *Alzheimer’s Research and Therapy*, reflect a growing emphasis on neuroscience and ophthalmology within this body of research.

Our findings reveal a strong internal collaboration network among organizations dedicated to studying ocular biomarkers for diagnosing AD. Rosa de Hoz, Elena Salobrar-García, Juan J. Salazar, Inés López-Cuenca, and José M. Ramírez are associated with Complutense University of Madrid in Spain, where they specialize in retina, ophthalmology, and vision science. Their most recent publication explored the potential relationship between changes in retinal layer thickness and AD, employing OCT to identify quantitative parameters linked to the disease (Sánchez-Puebla et al., 2024). In Italy, Claudio Del Percio, Giuseppe Noce, Claudio Babiloni, and Andrea Soricelli are affiliated with two institutions: Sapienza University of Rome and The University of Naples Parthenope, showcasing a strong co-authorship within the country. Their research centers on neurophysiology, brain function, cognitive neuroscience, and neurodegeneration. Their latest publication integrated imaging methods, such as PET-MRI and PET, to examine Resting-State Electroencephalographic Rhythms in Parkinson’s disease with cognitive impairment. Notably, Frank Verbraak has emerged as the leading author in this field, holding the highest number of publications, citations, and impact (H-index). His comprehensive review established that changes in retinal thickness may serve as indicators of neurodegeneration in AD, providing a theoretical foundation for the potential use of ocular markers in disease detection (den Haan et al., 2017).

### Research hotspots and frontiers

Our keyword analysis highlights the evolving trends in ocular markers associated with AD research from 2005 to 2024. In the initial period (2005–2010), the emphasis was primarily on fundamental diagnostic techniques. Keywords such as “spectroscopy” and “cerebrospinal fluid” emerged in 2005 and 2006, respectively, underscoring a focus on traditional diagnostic methods and fluid biomarkers. During these years, the term “*in vivo*” gained popularity, indicating a growing adoption of live imaging techniques in studies involving living organisms. From 2011 to 2015, research expanded significantly, with terms such as “degeneration,” “optic nerve,” and “apolipoprotein E” reflecting a growing interest in the pathological mechanisms of AD as they relate to ocular structures. By 2015, the emergence of “oxidative stress” as a prominent topic signaled a shift toward exploring the molecular mechanisms underlying Alzheimer’s pathology. In the mid-2020s (2015–2020), the focus shifted to neurodegenerative processes. Keywords like “nerve fiber layer,” “multiple sclerosis,” and “dysfunction” indicated an increasing interest in the clinical implications of these processes for ocular health. Concurrently, terms such as “CSF biomarkers” and “thickness” highlighted advancements in understanding CSF components and retinal thickness as potential diagnostic tools. In recent years (2020 onward), keywords such as “neurodegeneration” and “optical coherence tomography angiography” have gained prominence, indicating that high-precision imaging techniques, particularly OCTA, are becoming essential for detecting ocular biomarkers. This shift reflects a growing emphasis on advanced neurodegenerative marker analysis. Over time, topics like “cerebrospinal fluid” and “*in vivo*,” which were initially prominent in early research, have gradually been supplanted by newer techniques such as OCTA and concepts like “neurodegeneration,” illustrating rapid advancements in diagnostic technology.

Recent studies have highlighted OCTA as a non-invasive ocular imaging technique that effectively assesses the peripheral vascular system of the retinal microcirculation (Curro et al., 2024; Sun et al., 2024; Vagiakis et al., 2024). A study utilized OCTA and PET to investigate temporal changes in physiological parameters among patients with preclinical AD. In the Aβ + cohort, vessel density (VD) demonstrated non-significant incremental changes over time in both the macular and optic nerve head (ONH) regions. Conversely, the Aβ− cohort exhibited significantly elevated VD in both the ONH and macular regions. Furthermore, the Aβ++ group revealed markedly greater VD in both the inner and outer rings of the macula when compared to the Aβ+ and Aβ− groups. These variations in VD appear to emerge early in the progression of preclinical AD, underscoring distinct differences between the ONH and macular regions (Curro et al., 2024). Although a consensus regarding the utility of the foveal avascular zone as a biomarker remains elusive (Curro et al., 2024; Vagiakis et al., 2024), OCTA continues to show promise in differentiating AD from other neurodegenerative disorders (Sun et al., 2024).

In addition to the analysis of retinal structure, emerging detection technologies have enabled the examination of tear components and their movement parameters, which may contribute to the diagnosis of AD. Tears may provide a valuable source of biomarkers for AD due to their easy collection and accessibility. Elongation initiation factor 4E (eIF4E) has been found exclusively in the tears of AD patients, suggesting its potential to differentiate between various neurodegenerative diseases (Kenny et al., 2019). Additionally, the overall microRNA content is elevated in the tear samples of individuals with AD, with microRNA-200b-5p emerging as a particularly promising biomarker among the various individual microRNAs (Kenny et al., 2019). In the context of functional markers, eye movements serve as sensitive, low-cost, and non-invasive indicators for assessing cognitive changes (Chaitanuwong et al., 2023). Research indicates that the anti-sweep task reveals the most pronounced abnormalities in both early-stage and advanced AD, which are linked to frontal lobe dysfunction resulting from the neurodegenerative processes associated with the disease (Chehrehnegar et al., 2022). Furthermore, individuals with both early-stage and advanced AD demonstrate a significant increase in eye hopping latency and frequency errors when compared to cognitively healthy individuals (Opwonya et al., 2022).

Furthermore, the development of artificial intelligence (AI) in AD diagnosis has gradually emerged as a significant area of interest. Deep Learning (DL), a subset of AI, has been increasingly applied for the early detection of AD, which relies heavily on imaging for accurate diagnosis(Lagun et al., 2011; Cheung et al., 2022; Sun et al., 2022; Zuo et al., 2024). Cheung et al. utilized a substantial dataset of 12,949 fundus photographs collected from a diverse global population. This dataset comprised 7,351 images from 3,240 healthy individuals and 5,598 images from 648 patients diagnosed with AD. The study achieved an accuracy rate of 86.3% in detecting AD within its bilateral internal validation dataset. In the test dataset, the accuracy for AD detection varied, ranging from 79.6 to 92.1% (Cheung et al., 2022). Li et al. (2024) found significant differences in eye movement patterns between healthy controls and those with AD or mild cognitive impairment (MCI), suggesting potential biomarkers for early detection. Further, eye-tracking metrics have shown efficacy in clinical settings for monitoring cognitive decline in the elderly(Li et al., 2024). Opwonya et al. (2023) demonstrated that eye-tracking tasks, such as prosaccade and antisaccade paradigms, could distinguish cognitively normal individuals from those with MCI based on latency and error saccades. Integrating eye-tracking into diagnostic frameworks alongside other biomarkers may enhance understanding of the progression risk in individuals with subtle cognitive impairments. Moreover, the study found that eye-tracking performance correlates with amyloid plaque burden, suggesting its potential as a biomarker for early neurodegenerative disease detection (Opwonya et al., 2023).

Recent studies modeling eye movement data have also employed deep learning techniques to identify eye movement patterns associated with AD during three-dimensional visuospatial memory tasks (Sun et al., 2022; Zuo et al., 2024). Ophthalmic AI exhibits improved performance when utilizing multimodal retinal imaging techniques, rather than relying solely on fundus images (Chaitanuwong et al., 2023). Despite challenges such as limited dataset availability, a lack of data diversity, and inconsistent image quality, deep learning, due to its exceptional predictive capabilities, is still at the forefront of biomarker research in the detection of AD.

## Strengths and limitations

The study highlights several noteworthy strengths. First, the comprehensive bibliometric analysis offers an in-depth overview of research trends and key contributors in the field of Alzheimer’s disease (AD) ocular markers from 2005 to 2024. This longitudinal approach effectively captures the evolving landscape of this research area, revealing significant shifts and advancements over time. Additionally, the use of multiple bibliometric tools enhances the visual representation of collaborations, keyword co-occurrences, and emerging trends, providing a well-rounded understanding of the data.

However, there are several limitations that should be acknowledged. First, the study focuses exclusively on articles published in English, which may lead to the exclusion of relevant studies in other languages and introduce potential bias into the findings. Second, while relying on the Web of Science Core Collection (WoSCC) database is beneficial, it may have resulted in the omission of significant publications indexed in other databases, thereby constraining the scope of the analysis.

The bibliometric analysis highlights significant progress in ocular biomarker research for early AD diagnosis. Key institutions like Complutense University of Madrid and Sapienza University of Rome have made substantial contributions. Future research should focus on advanced imaging techniques like OCTA, tear component analysis, eye movement assessments, and artificial intelligence to enhance diagnostic accuracy and accessibility.

## Data Availability

The datasets presented in this article are not readily available because Web of Science Core Collection (WoSCC). Requests to access the datasets should be directed to Zhuoying Zhu, zhuzhuoying1991@163.com.
